# Relation between duration of the prodromal phase and renal damage in ANCA-associated vasculitis

**DOI:** 10.1186/s12882-017-0797-x

**Published:** 2017-12-29

**Authors:** Eline Houben, Stefanie L. Groenland, Joost W. van der Heijden, Alexandre E. Voskuyl, Hiëronymus J. Doodeman, Erik L. Penne

**Affiliations:** 1 Department of Internal Medicine, Northwest Clinics, Wilhelminalaan 12, 1815 JD Alkmaar, The Netherlands; 2 Department of Nephrology, VU University Medical Centre, Amsterdam, The Netherlands; 3 Department of Rheumatology, Amsterdam Rheumatology and Immunology Centre, VU University Medical Centre, Amsterdam, the Netherlands; 4 Northwest Academy, Northwest Clinics, Alkmaar, The Netherlands

**Keywords:** ANCA-associated vasculitis, Mortality, Prodromal phase, Proteinuria, Renal damage

## Abstract

**Background:**

In ANCA-associated vasculitis the acute phase of the disease is often preceded by prodromal symptoms. The aim of the present study was to analyze the relation between the duration of the prodromal phase and renal damage.

**Methods:**

Patients with ANCA-associated vasculitis and renal involvement from a retrospective single-center cohort were divided into two equal groups based on the duration of the prodromal phase. The prodromal phase was defined as the time between first vasculitis related symptoms and the date of diagnosis. Clinical characteristics at diagnosis and renal items on the vasculitis damage index at 6 months were compared between the two groups. In addition, the relation between a long prodromal phase and 3-year end-stage renal disease and mortality as a composite outcome was studied.

**Results:**

A total of 72 patients were included (age 64 ± 12 years; 74% male; 96% Caucasian). At diagnosis, in patients with a prodromal phase ≤22 weeks versus >22 weeks estimated glomerular filtration rate and proteinuria did not differ significantly (35 (interquartile range 50) versus 30 (50) ml/min *p* = 0.84; 75% versus 87%, *p* = 0.21 respectively). Furthermore, Birmingham Vasculitis Activity Scores were comparable (7 (3), *p* = 0.71). At 6 months, a long prodromal phase was associated with proteinuria (odds ratio 5.38, 95% confidence interval (CI) 1.47–19.62), but not with an estimated glomerular filtration rate ≤ 50 ml/min (odds ratio 0.89, 95% CI 0.33–2.37) in multivariable analyses. In addition, a long prodromal phase was associated with end-stage renal disease/mortality (hazard ratio 5.22, 95% CI 1.13–24.20).

**Conclusions:**

A long prodromal phase was associated with proteinuria and 3-year end-stage renal disease/mortality, but not with a reduced renal function at 6 months. These results underline the importance of an early diagnosis in ANCA-associated vasculitis patients in order to improve renal outcomes.

## Background

Antineutrophil cytoplasmic antibody (ANCA)-associated vasculitis (AAV) is an autoimmune disease characterised by inflammation of the blood vessel wall. AAV comprises granulomatosis with polyangiitis (GPA), microscopic polyangiitis (MPA) and eosinophilic granulomatosis with polyangiitis (EGPA) [1].

The course of AAV is variable, however most patients experience a first, prodromal phase with aspecific symptoms such as fatigue, myalgia and flu-like upper respiratory tract inflammation [2]. The duration of the prodromal phase is variable and can be weeks up to even years [3]. Most patients proceed to a more acute phase with severe symptoms such as rapidly progressive glomerulonephritis, involvement of the central nervous system and pulmonary hemorrhage [2, 4]. Diagnosing AAV during the prodromal phase is challenging, since symptoms are often aspecific, leading to both patient and doctors delays [4].

It is currently unclear to what extent renal damage already occurs in the prodromal phase. A first step in the improvement of care would be to study the influence of prodromal phase length on renal damage. Only a few studies have addressed the relation between time to diagnosis and prognosis. These studies, however, were either inconclusive or included patients that were diagnosed in the past century [5–8]. It is unknown to what extent these results still apply to current daily practice. The aim of the present study was to explore whether patients with a long prodromal phase had more active disease at presentation in comparison with patients with a short prodromal phase. Furthermore, the association between a long prodromal phase and renal damage and mortality was studied.

## Methods

In the Northwest Clinics, a teaching hospital in The Netherlands, clinical characteristics of all consecutive anti-proteinase 3 (PR3) or anti-myeloperoxidase (MPO) ANCA positive patients between February 1st 2005 and February 1st 2015 were retrospectively collected from the medical files [9]. Patients were eligible if they had a clinical diagnosis of AAV in consistence with the Chapel Hill Consensus Conference guidelines [1]. In order to study renal damage secondary to vasculitis, patients were only included if renal involvement was present at diagnosis. Renal involvement was defined, in accordance with the Birmingham Vasculitis Activity Score specific for Wegener’s Granulomatosis (BVAS/WG), as a rise in serum creatinine of >30% and/or erythrocyturia (≥10 red blood cells per high-power field and/or red blood cell casts) [10]. Patients were classified into GPA, MPA or EGPA in accordance with the European Medicines Agency algorithm [11].

Patients with AAV and renal involvement were retrospectively divided into two equal groups based on a long and short prodromal phase. Prodromal phase was defined as the time between first AAV-associated signs or symptoms, as reported in the medical file at the first hospital visit, and the date of diagnosis. The median was used as the cut-off value.

Disease activity (BVAS/WG), treatment regimens and other clinical characteristics at diagnosis were compared between the two groups. Renal function before the onset of symptoms was collected, if available. In order to assess renal damage and not active vasculitis, renal items on the vasculitis damage index (VDI) [12] were collected 6 months after diagnosis. Six months is generally the maximum duration of remission-induction therapy and therefore most of the renal symptoms at 6 months can be considered as chronic damage [13]. Renal items on the VDI comprise an estimated glomerular filtration rate (eGFR) ≤ 50 ml/min (calculated by the Chronic Kidney Disease Epidemiology Collaboration (CKD-EPI) equation), end-stage renal disease (ESRD) and/or proteinuria ≥0.5 g/24 h [12–14]. If 24 h urine was not available, proteinuria was defined as ≥300 mg/l protein in a urine sample. Furthermore the relation between the prodromal phase and mortality or end-stage renal disease (ESRD) at 3 years as a composite outcome was studied.

### Statistical analysis

Characteristics at diagnosis and at 6 months were compared between patients with a short and long prodromal phase with Chi-square tests, unpaired Student’s t-tests or Mann Whitney U tests as appropriate.

The association between prodromal phase length and renal damage at 6 months was tested with univariable logistic regression models. In order to correct for confounding factors: sex, age, type of AAV and disease activity at diagnosis, multivariable logistic regression models were developed. Confounding factors were selected in a forward selection procedure with a limit of 10% change in effect size using basic logistic regression models with only short/long prodromal phase as independent variable and eGFR, proteinuria or ESRD as dependent variables. The confounder with the largest change in effect size of the determinant was included in the new model. The selection procedure was then repeated on the new model, using the remaining covariables. The procedure was stopped after none of the covariables changed the effect size by more than 10% or after reaching the maximum amount of confounders defined as 10% of the number of outcomes.

In order to test the association between prodromal phase length and time to ESRD/mortality as a composite outcome, Cox regression analysis was used. Patients were followed up to death, ESRD or 3 years, whichever came first. A log minus log plot was visually inspected in order to judge if the proportional hazard assumption was valid. A Kaplan-Meier plot was used to visualise the association. In view of the limited number of events, separate bivariable cox regression analyses were used to adjust for the effect of age, sex, GPA, or BVAS/WG. A two-sided *p*-value <0.05 was considered statistically significant. Statistical analyses were conducted using Statistical Package for Social Sciences (SPSS®) version 20.0.

## Results

### Patients

**Fig. 1:**
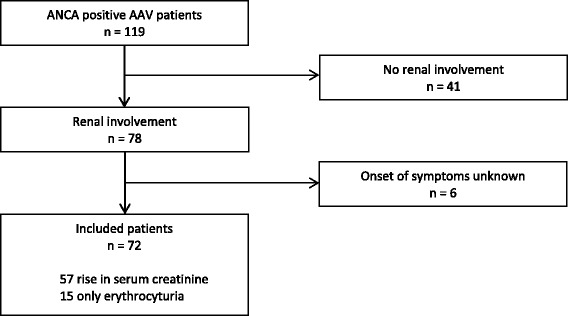
Flow diagram of patient selection

A total of 78 patients with AAV and renal involvement were identified. In six patients the onset of symptoms was missing in the medical records; these patients were excluded (Fig. 1). In the remaining 72 patients the median time from first AAV-related signs or symptoms to diagnosis was 22 weeks (interquartile range (IQR) 47 weeks). At diagnosis, the mean age ± standard deviation (SD) did not differ between patients with a prodromal phase ≤22 weeks and patients with a prodromal phase >22 weeks. However, patients with a shorter prodromal phase tended to be slightly older (66 ± 11 versus 61 ± 13 years, *p* = 0.10). GPA was more often diagnosed in patients with a long prodromal phase, whilst MPA was more frequently diagnosed in patients with a short prodromal phase. eGFR and proteinuria at diagnosis did not differ between the two groups. Anti-PR3 positivity was more frequent than anti MPO positivity (58% versus 42%). As expected, a higher percentage of anti-PR3 positive patients (79%) were classified as GPA as compared with anti-MPO positive patients (43%), *p* = 0.002.

Most patients (75%) received a cyclophosphamide based induction regimen. In patients with a prodromal phase ≤22 weeks 28 patients (78%) received cyclophosphamide, seven patients only glucocorticoids (19%) and one patient was treated with glucocorticoids and azathioprine (3%) at diagnosis. In patients with a prodromal phase >22 weeks 26 (72%) were treated with cyclophosphamide, five with glucocorticoids (14%) and five other patients with either rituximab, methotrexate, azathioprine or mycophenolate mofetil. Maintenance regimens did not differ significantly between the two groups. In patients with a prodromal phase ≤22 weeks 29 (81%) were treated with a combination of azathioprine and glucocorticoids, as compared with 26 patients (72%) in the other group. Other maintenance regimens consisted of glucocorticoids or mycophenolate mofetil. In patients with a prodromal phase >22 weeks, four patients had died before the initiation of maintenance treatment. The full list of characteristics at diagnosis is provided in Table 1.

**Table 1:** Clinical characteristics at diagnosis of patients with ANCA-associated vasculitis and renal involvement with a short and long prodromal phase

Clinical characteristics at diagnosis				
	Total *n* = 72	*T* ≤ 22 weeks *n* = 36	*T* > 22 weeks *n* = 36	*p*-value
Male sex	74	70	78	0.42
Age, mean ± sd	64 ± 12	66 ± 11	61 ± 13	0.10
Caucasian	96	100	92	0.37
PR3 ANCA	59	56	61	0.63
MPO ANCA	42	44	39	0.63
ANCA-titre ≥4 times cut-off value	85	94	75	0.03
Induction therapy cyclophosphamide and glucocorticoids	75	78	72	0.49
Maintenance therapy azathioprine and glucocorticoids	76	81	72	0.23
Organ involvement^a^				
Pulmonary	44	50	39	0.34
ENT	43	31	56	0.03
Arthritis/arthralgia	40	31	50	0.09
Neurologic	31	25	36	0.31
Skin and soft tissue	15	14	17	0.74
Type of AAV				
GPA	64	50	78	0.05
MPA	32	44	19	
EGPA	4	6	3	
Renal biopsy proven	44	44	44	1.00
BVAS/WG at diagnosis, median (IQR)	7 (3)	7 (3)	7 (3)	0.71
Erythrocyturia^b^	75	81	69	0.63
Renal insufficiency^c^	79	78	81	0.77
eGFR (ml/min), median (IQR)^d^	34 (50)	35 (50)	30 (51)	0.84
Proteinuria^e^	81	75	87	0.21
CCI, median (IQR)	0 (1)	0 (1)	0 (1)	0.67
FFS, median (IQR)	2 (2)	2 (2)	1 (2)	0.12

Data expressed as percentage unless otherwise stated

*ANCA* antineutrophil cytoplasmic antibody, *BVAS/WG* Birmingham Vasculitis Activity Score specific for Wegener’s Granulomatosis, *CCI* Charlson Comorbidity Index, *EGPA* eosinophilic granulomatosis with polyangiitis, *FFS* five factors score, *GPA* granulomatosis with polyangiitis, *IQR* interquartile range, *MPA* microscopic polyangiitis, *MPO* myeloperoxidase, *PR3* proteinase 3, *sd* standard deviation; *T ≤ 22 weeks* prodromal phase ≤22 weeks, *T > 22 weeks* prodromale phase >22 weeks

^a^Organ involvement at time of diagnosis, in accordance with BVAS/WG

^b^Erythrocyturia was defined as ≥10 erythrocytes per high power field

^c^Renal insufficiency was defined as a rise in creatinine of >30%

^d^eGFR calculated by the Chronic Kidney Disease Epidemiology Collaboration equation

^e^Proteinuria was defined as ≥0.5 g/24 h or ≥300 mg/l protein in a urine sample

### Renal damage at six months

Before the onset of symptoms median eGFR was tested in 36 of 72 patients and was 94 (35) versus 88 (31) ml/min (*p* = 0.62) in patients with a prodromal phase ≤22 weeks versus >22 weeks. At diagnosis median eGFR was 35 (50) versus 30 (51) ml/min, *p* = 0.84. At 6 months, median eGFR and mean systolic blood pressure did not differ between patients with a prodromal phase ≤22 weeks versus >22 weeks (eGFR 45 (32) versus 50 (46) ml/min, *p* = 0.70 and blood pressure 133 ± 19 versus 131 ± 20 mmHg, *p* = 0.82). In a multivariable analysis the percentage of patients with an eGFR ≤50 ml/min did not differ significantly between the two groups (odds ratio (OR) 0.89, 95% confidence interval (CI) 0.33–2.37). Proteinuria at 6 months was more often present in patients with a long prodromal phase (OR 5.38, 95% CI 1.47–19.62), see Table 2.

**Table 2:** Relation between renal damage at six months and prodromal phase length

Renal damage at 6 months						
			Univariable analysis		Multivariable analysis	
	T ≤ 22 weeks	T > 22 weeks	OR (95% CI)	*p*-value	OR (95%CI)	*p*-value
eGFR ≤50 ml/min at 6 months, no (%)	21/35 (60)	19/36 (53)	0.75 (0.29–1.91)	0.54	0.89(0.33–2.37)^a^	0.52
Proteinuria at 6 months, no (%)	12/28 (43)	22/29 (76)	4.19 (1.14–13.01)	0.01	5.38 (1.47–19.62)^b^	0.002
ESRD, no (%)	3 (8)	4 (11)	1.38 (0.29–6.63)	0.69	n/a	

*BVAS/WG* Birmingham Vasculitis Activity Score specific for Wegener’s Granulomatosis, *ESRD* end-stage renal disease, *GPA* granulomatosis with polyangiitis, *n/a* not applicable, *T ≤ 22 weeks* prodromal phase ≤22 weeks, *T > 22 weeks* prodromale phase >22 weeks

^a^adjusted for age. The variables sex, GPA versus no GPA and BVAS/WG did not meet the confounder criteria

^b^adjusted for GPA versus no GPA. The variables age, sex and BVAS/WG did not meet the confounder criteria. Multivariable logistic regression analysis for ESRD at 6 months was not performed due to a low number of events in both groups

### Mortality and end-stage renal disease

**Fig. 2:**
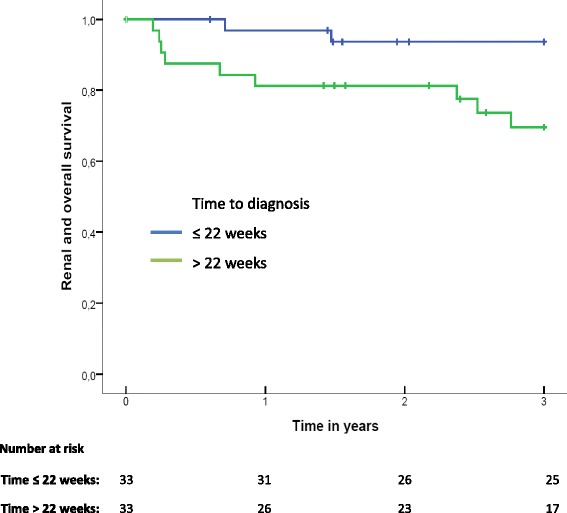
Kaplan-Meier curve of renal and overall survival in ANCA-associated vasculitis patients with a prodromal phase ≤22 weeks and >22 weeks

Within 3 years after diagnosis, nine patients (13%) developed ESRD, of which three patients in the group with a prodromal phase ≤22 weeks and six patients with a prodromal phase >22 weeks. In patients with a prodromal phase ≤22 weeks three patients died, in comparison to eight patients with a long prodromal phase. In Fig. 2 the Kaplan Meier curve illustrates the incidence of ESRD/mortality in the two groups. The graph of the log of the cumulative hazard function of both groups appeared parallel (data not shown), suggesting no violation of the proportional hazards assumption. After separate correction for age, sex, GPA versus no GPA and BVAS/WG a long prodromal phase remained significantly associated with 3 year ESRD/mortality, see Table 3.

**Table 3:** Bivariable cox regression analyses for the relation between prodromal phase length and 3 year ESRD/mortality as a composite outcome

Relation between prodromal phase length and ESRD/mortality		
Prodromal phase >22 weeks	HR (95% CI)	*p*-value
Univariable analysis	5.22 (1.13–24.20)	0.02
Analysis adjusted for confounders:		
Adjusted for age	8.63 (1.77–42.13)	0.01
Adjusted for male sex	5.85 (1.26–27.29)	0.02
Adjusted for GPA versus no GPA	5.92 (1.17–30.00)	0.03
Adjusted for BVAS/WG	5.48 (1.18–25.39)	0.03

Six patients were excluded from the survival analyses since they had already started renal replacement therapy before the diagnosis AAV and were therefore not at risk. Of these patients, 3 had a prodromal phase ≤22 weeks and 3 patients had a prodromal phase >22 weeks

*BVAS/WG* Birmingham Vasculitis Activity Score specific for Wegener’s Granulomatosis, *CI* confidence interval, *ESRD* end-stage renal disease, *GPA*, granulomatosis with polyangiitis, *HR* hazard ratio

## Discussion

In the present study the relation between the duration of the prodromal phase and renal damage in AAV patients was explored. At diagnosis, disease activity, proteinuria and kidney function were comparable for patients with a prodromal phase ≤22 weeks versus >22 weeks. At 6 months, kidney function was still similar in both groups. However, proteinuria was more often present in patients with a long prodromal phase. Furthermore a long prodromal phase was associated with a higher risk in time of ESRD and mortality as a composite outcome.

Our data showed that a long duration of the prodromal phase, in the absence of immunosuppressive therapy, leads to more severe renal outcomes. Renal involvement was present in all included patients at diagnosis and may have been smouldering in the weeks, months or even years prior to diagnosis. It is remarkable that at diagnosis disease activity and renal involvement appeared equally severe in both groups. This supports the hypothesis that patients are generally diagnosed in the acute phase of the disease, when symptoms are pronounced. However, at 6 months after diagnosis patients with a longer prodromal phase had more damage as defined by the presence of proteinuria. This suggests that if the prodromal phase exists for a longer time, renal damage becomes more irreversible. Proteinuria is an important marker for kidney damage and a strong predictor for progression of renal disease [15]. A higher burden of proteinuria may have predisposed to the development of ESRD/mortality later in the disease course [16, 17].

Previous studies that addressed the relation between the duration of the prodromal phase and prognosis reported conflicting results. Two previous studies analysed the relation between time to diagnosis and survival, among which, one study with 50 GPA patients and one study with 595 MPA and EGPA patients. In both studies no association was found between the duration of the time to diagnosis and survival [5, 8]. One older study found an association between the time to diagnosis and survival and a VDI ≥ 1 in GPA patients [6]. So far only one study has focused on renal damage in particular. In this study an association was found between a longer prodromal phase and ESRD [7]. The present study focused on survival, ESRD and renal damage at 6 months. A strength of the current study is that all patients with AAV and renal involvement were included, instead of only those with a renal biopsy proven diagnosis. This results in a realistic representation of clinical practice.

So far no study has assessed the potential effect of lead-time bias in the association. Lead time bias means that patients with a diagnosis at an earlier stage do not live longer, but only live longer with the established disease due to an earlier detection. The fact that in our cohort both groups presented with a comparable disease activity and comparable severity of renal symptoms, suggests that patients with a longer prodromal phase were not simply diagnosed at a later stage in the disease. Nevertheless, we cannot rule out that lead time bias does play some role in the observed association. Depending on the extent of this effect, our results should be adjusted for lead-time bias. Considering the different disease courses in both groups, it is currently uncertain to what extent our final results should be corrected for lead-time bias.

This study is limited by its retrospective design. Although this design is inevitable in answering our questions, this may account for a bias in reported symptoms in the medical records, which could lead to an underscore of the BVAS/WG and led to the inability to calculate the entire VDI at 6 months. Furthermore, the investigators depended on the onset of symptoms as reported in the medical records, which is limited by the variety in precise recall by patients and accuracy of history taking by doctors. The quality of the medical documentation at a first visit was relatively high, with a documentation of the onset of symptoms in 72 out of 78 patients. However, patients may not mention symptoms that they do not correlate to their current problem and physicians may not ask for all vasculitis related symptoms. Despite this limitation, the time between the onset of symptoms and diagnoses appeared to be in the same range as in previous studies [3, 5]. The Northwest Clinics is a teaching hospital, that covers a large area in the North of the Netherlands. Therefore, most of the included patients were both diagnosed and followed up in the same center. However, our study lacks data on possible treatments carried out by family physicians, which may have affected prodromal phase length. Due to the rarity of the disease, our cohort exists of a fairly small number of patients, which resulted in a limited number of events and impaired the evaluation of potential confounders.

## Conclusions

Our study confirmed that the prodromal phase in AAV patients is long and that the prognosis is poor in both groups. Proteinuria and ESRD/mortality was increased in patients with a longer duration of prodromal symptoms. These findings stress the importance of an early diagnosis in AAV, even though this can be challenging when symptoms are aspecific. Education in primary and secondary care on the different aspects of AAV could increase awareness, so that patients could be referred earlier to an internist or rheumatologist. Furthermore, simple and well-established diagnostic criteria could be helpful in an earlier diagnosis. The European League Against Rheumatism and the American College of Rheumatology are currently developing and validating new diagnostic and classification criteria for primary systemic vasculitis [18]. This could eventually lead to a shorter time to diagnosis and better renal outcomes.

## Abbreviations

AAV: ANCA-associated vasculitis
ANCA: Antineutrophil cytoplasmic antibody
BVAS/WG: Birmingham Vasculitis Activity Score specific for Wegener’s Granulomatosis
CKD-EPI: Chronic Kidney Disease Epidemiology Collaboration
eGFR: Estimated glomerular filtration rate
EGPA: Eosinophilic granulomatosis with polyangiitis
ESRD: End-stage renal disease
GPA: Granulomatosis with polyangiitis
IQR: Interquartile range
MPA: Microscopic polyangiitis
MPO: Myeloperoxidase
PR3: Proteinase 3
SD: Standard deviation
SPSS®: Statistical Package for Social Sciences
VDI: Vasculitis Damage Index
